# Advancing AI models for enhanced melanoma diagnosis

**DOI:** 10.1111/jdv.70023

**Published:** 2025-10-25

**Authors:** François Kuonen, Olivier Gaide

**Affiliations:** ^1^ Department of Dermatology and Venereology Lausanne University Hospital Center Lausanne Switzerland

Melanoma is one of the most aggressive skin cancers, its prognosis closely tied to early and accurate diagnosis. Improving diagnostic accuracy thus has the promise of lowering morbidity and mortality of melanoma, provided most patients can rapidly benefit from it. Over the past decade, artificial intelligence (AI)–assisted diagnostic tools have advanced markedly in classifying dermoscopic images, achieving dermatologist‐level performance.^1^ These gains have been driven by larger annotated datasets, refinements in convolutional neural network (CNN) architectures and greater computational capacity.

Despite these advances, clinical integration of AI is limited due to the lack of a robust demonstration of diagnostic performance across diverse populations in clinically relevant settings. But before large prospective studies are initiated, careful selection of model architecture (i.e. selecting the most promising AI tool) is crucial. Individual studies vary widely in design, patient demographics, imaging modalities and reference standards, limiting generalizability.^2^ Moreover, few have evaluated performance across a broad range of skin phototypes, lesion sites, co‐existing conditions and image quality.^3^ In this context, the systematic meta‐analysis by Ertürk Zararsiz et al. is timely, pooling data from multiple independent studies to generate more reliable estimates of diagnostic performance.^4^ The authors appropriately focus on the area under the receiver operating characteristic curve (AUC), a threshold‐independent metric well suited for cross‐study comparison.^5^ Their results are compelling: a pooled AUC of 0.96 for AI‐based melanoma detection strongly supports the potential of AI‐assisted dermatoscopic diagnosis.

Different algorithmic approaches—from classical machine learning (ML) to deep learning (DL) and, more recently, *hybrid models*—can differ in accuracy, robustness and generalisability. *Hybrid models* refer to architectures combining distinct algorithmic components, for example a CNN for image feature extraction paired with a support vector machine (SVM), or CNNs integrated with additional deep learning modules. Such designs exploit complementary strengths: CNNs capture high‐dimensional image features, while secondary classifiers can refine decision boundaries, mitigate overfitting and integrate heterogeneous inputs. Consistently, the hybrid subgroup achieved the highest pooled performance (AUC = 0.98). Notably, the performance gain from hybrid models exceeded the incremental improvements typically seen when moving from ML to DL, or from shallow CNNs to deeper convolutional architectures (DCNNs), suggesting that combining heterogeneous approaches may yield greater benefits than single‐paradigm refinements.

Several limitations however merit consideration. Study heterogeneity in design, image acquisition and reference standards may introduce bias. *Hybrid* models are also relatively recent, and the studies evaluating them may have benefitted from larger datasets, improved annotation quality and more advanced computing resources. Many included dermatoscopic datasets that might have been curated or that excluded diagnostically challenging cases, most likely inflating performance relative to real‐world practice. Furthermore, while AUC is a robust indicator of discriminative ability, it does not guarantee clinical utility until proven by prospective validation. Finally, this meta‐analysis focused on AI models alone. Prior research strongly suggests that combining AI outputs with dermatologist expertise can further enhance diagnostic accuracy, particularly in borderline or atypical presentations.^2^ Such human–AI collaboration, augmented intelligence, offers additional gains beyond the performance of even the best standalone AI models.

In summary, this meta‐analysis provides compelling evidence that AI—particularly *hybrid* models—currently achieves very high diagnostic accuracy for melanoma detection from dermatoscopic images. However, realizing its full clinical potential will require rigorous prospective validation and careful integration into clinical workflows, including combination with expert human judgement, a synergy that offers the greatest promise for enabling earlier melanoma detection and improving patient outcomes.

## FUNDING INFORMATION

No funding supported the work.

## CONFLICT OF INTEREST STATEMENT

None.

## ETHICS STATEMENT

Not applicable.

## Data Availability

Data sharing not applicable to this article as no datasets were generated or analysed during the current study.

## References

[1] Esteva A , Kuprel B , Novoa RA , Ko J , Swetter SM , Blau HM , et al. Dermatologist‐level classification of skin cancer with deep neural networks. Nature. 2017;542(7639):115–118. 10.1038/nature21056 28117445 PMC8382232

[2] Tschandl P , Rinner C , Apalla Z , Argenziano G , Codella N , Halpern A , et al. Human–computer collaboration for skin cancer recognition. Nat Med. 2020;26(8):1229–1234. 10.1038/s41591-020-0942-0 32572267

[3] Daneshjou R , Vodrahalli K , Novoa RA , Jenkins M , Liang W , Rotemberg V , et al. Disparities in dermatology AI performance on a diverse, curated clinical image set. Sci Adv. 2022;8(32):eabq6147. 10.1126/sciadv.abq6147 35960806 PMC9374341

[4] Ertürk Zararsız G , Yerlitaş Taştan SI , Çelik Gürbulak E , Erakcaoğlu A , Yılmaz Işıkhan S , Demirbaş A , et al. Diagnosis melanoma with artificial intelligence systems: a meta‐analysis study and systematic review. J Eur Acad Dermatol Venereol. 2025;39(11):1912–1922. 10.1111/jdv.20781 PMC12553134 40476369

[5] Mandrekar JN . Receiver operating characteristic curve in diagnostic test assessment. J Thorac Oncol. 2010;5(9):1315–1316. 10.1097/JTO.0b013e3181ec173d 20736804

